# Reduced Lung Function in a Chronic Asthma Model Is Associated with Prolonged Inflammation, but Independent of Peribronchial Fibrosis

**DOI:** 10.1371/journal.pone.0001575

**Published:** 2008-02-06

**Authors:** Cordula Koerner-Rettberg, Sandra Doths, Anke Stroet, Jürgen Schwarze

**Affiliations:** 1 Children's Hospital, Ruhr University of Bochum, Bochum, Germany; 2 Centre for Inflammation Research, University of Edinburgh, Queens Medical Research Institute, Edinburgh, United Kingdom; Centre de Recherche Public-Santé, Luxembourg

## Abstract

**Background:**

In asthma, mechanisms contributing to chronicity remain to be determined. Recent models of sensitisation with prolonged airway allergen challenges reproduce typical features of chronic asthma. However, the interplay between inflammation, structural changes and lung function is poorly understood. This study was performed to delineate functional, structural and immunological airway changes after cessation of long term challenges to elucidate factors contributing to the development of prolonged lung function changes.

**Methodology/Principal Findings:**

Mice sensitised systemically were consecutively challenged intranasally with ovalbumin for two or eight weeks. After the end of challenges, lung function, airway inflammation, features of airway remodelling, local T-cell cytokines and systemic ovalbumin-specific antibodies were monitored. Long term challenges resulted in airway hyperresponsiveness lasting 2 weeks and reduced baseline lung function for 6 weeks after their cessation. In contrast, these changes resolved within one week after short term challenges. Prolonged transforming growth factor beta (TGF-β)1 production and marked peribronchial fibrosis were only induced by long term challenges. Importantly, fibrosis became apparent only after the onset of lung function changes and outlasted them. Further, long term challenges led to prolonged and intense airway inflammation with marked lymphocytosis, but moderate eosinophilia, sustained IL-5 production and ovalbumin-specific IgG2a antibodies, the latter suggesting a Th1 component to the immune response. In contrast, following short term challenges airway inflammation was dominated by eosinophils and associated with a strong, but transient IL-13 response.

**Conclusions:**

Prolonged lung function changes after long term allergen challenges seem to develop and resolve independently of the persistent peribronchial fibrosis. They are more closely associated with intense airway inflammation, marked lymphocytosis, prolonged IL-5 and TGF-β1 production in the airways and a Th1 immune response.

## Introduction

Asthma now affects more than 10% of children in industrialized countries [1] and results in considerable morbidity and mortality. Current asthma management does not prevent chronic disease. We therefore need to understand the pathogenic mechanisms leading to chronicity. Defining the impact of chronic structural changes on lung function is of particular interest.

Mouse models of allergic airway sensitisation with short term allergen challenges have elucidated mechanisms of acute inflammatory and functional responses like airway eosinophilia and transient airway hyperresponsiveness (AHR) (reviewed by Kumar [2]) but failed to induce characteristic features of chronic asthma, such as airway wall remodelling, including sub-epithelial fibrosis [3] and muscular hypertrophy [4], and long term changes in lung function, all of which can be seen in models with prolonged airway challenges [5]–[14]. However, the protocols employed vary significantly, and only few of the long term studies investigated the persistence of changes after final allergen contact yielding conflicting results especially regarding persistence of impaired lung function [5], [9], [13]. Thus, the nature of interactions between airway remodelling and impaired lung function remains poorly defined.

Predominance of Th2 cytokines in murine models of acute allergic airway inflammation is well established [15] and important roles of IL-5 in induction of airway eosinophilia and of IL-5, IL-4 and IL-13 in the development of AHR [16], [17] have been defined. More recently, profibrotic roles for IL-13 and IL-4 [18], [19] in airway remodelling have emerged. Data comparing T-cell responses between models with short term and chronic allergen challenges are scarce. In atopic dermatitis, a shift from an initial Th2 response to a Th1 response in chronic disease has been reported [20]. There is also some evidence supporting a contribution of Th1 cytokines to the immune response in severe human asthma [21], [22]. Interestingly, evidence of Th1 responses with IFN-γ production has also been found recently in a murine model of chronic airway sensitisation [9].

Here, we report a sensitisation model with long term airway allergen challenges which induce typical features of chronic asthma including protracted changes in lung function, airway inflammation, mucus hyperplasia and peribronchial fibrosis. To delineate factors contributing to these chronic changes we compared immunological, functional and structural consequences after the cessation of long and short term challenges.

## Materials and Methods

### Animals

Female BALB/c mice, 6–8 weeks of age, from Charles River Deutschland (Sulzfeld, Germany) were used under protocols approved by Regierungspräsidium Arnsberg (NRW, Germany).

### Experimental protocol

Mice were sensitised on days 1 and 7 by intraperitoneal injection of 20 µg ovalbumin (OVA) (Sigma-Aldrich, Taufkirchen, Germany) emulsified in 2.25 mg aluminium hydroxide (AlumImuject; Pierce, Rockford, Ill) in 100 µl. Mice were challenged intranasally with 40 µl of 1% OVA in PBS under light anaesthesia with xylazine (BayerVital, Leverkusen, Germany)/ketamine (CuraMED Pharma, Karlsruhe, Germany). Controls were treated intraperitoneally with PBS, emulsified in aluminium hydroxide, and intranasally with sterile PBS. Two different challenge protocols were used: once weekly airway challenges for 2 weeks (short term) or for 8 weeks (long term). 48 hours, 2, 4, and 8 weeks after the final airway challenge lung function was determined. 24 hours after lung function tests, mice were sacrificed for harvest of bronchoalveolar lavage (BAL) fluid, lungs and serum. Blood was drawn by tail vein puncture prior to cervical dislocation.

### Lung function measurements

Baseline lung function and airway responsiveness to increasing concentrations of methacholine (MCh) (Sigma-Aldrich) were assessed using a single-chamber, whole-body plethysmograph (Buxco Electronics Inc, Troy, New York, USA) as described [23]. Briefly, unrestrained, spontaneously breathing mice were placed in plethysmographs and challenged with aerosols of PBS and increasing concentrations of MCh. At baseline after PBS-exposure and following each provocation, enhanced pause (Penh) values were monitored for 4 min.

### Lung histology

Lungs were fixed with 4% paraformaldehyde prior to embedding in paraffin. 5 µm sections were stained with haematoxylin/eosin to assess inflammatory infiltrates, with Alcian Blue Periodic Acid -Schiff (PAS) to detect mucin in goblet cells, and with Masson's trichrome to determine the amount of peribronchial collagen (all dyes from Merck, Darmstadt, Germany). Sections were analysed using a Zeiss Axioplan 2 imaging microscope, Axiocam camera and Axiovision 4.3 software (Zeiss, Jena, Germany). Airway inflammation was quantified in the peribronchial region of 6–8 different medium-sized bronchi per slide at ×20 magnification, using a semi-quantitative scoring system with a grading scale from 0 (no inflammation) to 4 (very severe inflammation). Goblet cell hyperplasia was determined by enumerating PAS-positive cells in airway epithelium at ×40 magnification. Collagen deposition (green stain) was quantified in peribronchial areas in sections stained with Masson's trichrome using AnalySis 3.2 software (Software Imaging Systems, Münster, Germany) by determining the ratio of green area per total tissue/matrix area (green/100-non-stained).

### Assessment of BAL inflammatory cells

Total leukocyte numbers in the BAL fluid were counted in a Neubauer chamber. Following cyto-centrifugation (Cytospin, Shandon Inc., Pittsburgh, PA) and staining with Haema Schnellfärbelösung, (Labor und Technik, Eberhard Lehmann, Berlin, Germany), at least 300 cells per slide were differentiated by a blinded investigator using standard haematological criteria.

### Measurement of cytokines in BAL fluid

Cytokine levels in BAL fluid were measured by ELISA for IL-4, IL-10 and IL-12 (OptEIA, Becton Dickinson, Heidelberg, Germany) as well as for IL-13 and activated TGF-β1 (Quantikine Sandwich ELISA, R&D Systems, Wiesbaden, Germany) according to the manufacturer's directions. In IL-5 and IFNγ ELISA the following antibodies were used: Purified rat anti-mouse IL-5 (TRFK5) for coating and biotin rat anti-mouse IL-5 (TRFK4) for detection, and rat anti-mouse IFNγ (XMG1.2) purified and biotinidated for coating and detection (all Pharmingen, Becton Dickinson, Heidelberg, Germany). The detection limits were 7 pg/ml for IL-4, 15 pg/ml for IL-5, 8pg/ml for IL-10, 80 pg/ml for IL-12p70, 5 pg/ml for IL-13, 20 pg/ml for IFNγ and 30pg/ml for activated TGF-β1.

### Flow cytometry

Lungs were minced, digested with collagenase (Sigma-Aldrich) for 30 min, and washed. Mononuclear cells were isolated by Ficoll (Biochrom AG, Berlin, Germany) gradient. For intracellular cytokine staining, lung cells were stimulated for 4 hours with PMA (30 ng/ml), and ionomycin (300 ng/ml) in the presence of brefeldin A (10 µg/ml) (all from Sigma-Aldrich). Following fixation and permeabilization, cells were stained with the appropriate antibodies or isotype controls. A FACScan analyser (Becton Dickinson) was used for data acquisition and Cellquest software (Becton Dickinson) for analysis. The following antibodies (all Becton Dickinson) were used: anti-CD3 (145-2C11) FITC-conjugated; anti-IL-4 (11B11), anti-IL-5 (TRFK5), and anti-IFNγ (XMG1.2) PE-conjugated, rat IgG2a (R35-95), rat IgG2b (A95-1), rat IgG1 (R3-34), mouse IgG2b (MPC-11) and hamster IgG (A19-3) were used as isotype controls.

### Measurement of OVA-specific antibodies in the serum

Serum levels of OVA-specific IgE, IgG1, and IgG2a were measured by ELISA as described. Briefly, 96-well-plates (Greiner bio-one, Frickenhausen, Germany) were coated with OVA (5 µg/ml) (Sigma-Aldrich) in carbonate buffer and washed. Then serum samples were added and plates were washed again. For IgE detection, biotinylated anti-IgE antibody (R35-72, Becton Dickinson) was used with extravidin-POD (Sigma-Aldrich) and TMB as substrate. OVA-specific IgG1 and IgG2a were detected with alkaline phosphatase-labeled anti-IgG1 (X56) or anti-IgG2a (R19-15) antibodies (both Becton Dickinson) respectively and pNPP substrate (Sigma-Aldrich). OVA-specific antibody titres were related to an internal, pooled standard and expressed as internal units (IU)/ml. The detection limit for OVA-specific IgE was 2,0 IU/ml, for OVA-specific IgG1 and –IgG2a 0,001 IU/ml.

### Statistical analysis

Data were compared using GraphPad Prism 4.02 (GraphPad Software, San Diego, USA). Mann-Whitney test was used for comparison of 2 groups and Kruskal-Wallis test with Dunn post hoc test were used for comparisons of more than 2 groups. P values for significance were set at 0.05 except for flow cytometry data and Penh data where p values of <0.01 were regarded as significant. Values are expressed as mean±SEM for all measurements.

## Results

### Lung function

**Figure 1:**
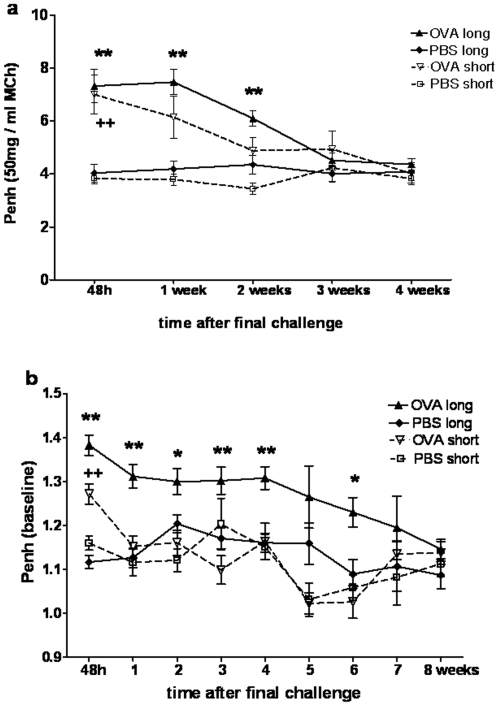
Lung function following long and short term airway challenges. (a) Following OVA sensitisation and short or long term OVA challenges or after sham sensitisation and long or short term PBS challenges, airway responsiveness to MCh was assessed 48 hours after final airway challenge and then weekly for 8 weeks. Mean±SEM of Penh values at 50 mg/ml MCh are shown for the first 4 weeks only from 3 independent experiments (n≥12). No differences between groups were detected after week 4. (b) Baseline Penh values were assessed during the same measurements. Mean±SEM from 5 independent experiments are illustrated (n≥15). Significant differences: * long term OVA challenges versus PBS control, +short term OVA challenges versus PBS control, levels of significance: */+ p<0,01, **/++ p<0,001.

Following short term as well as long term OVA challenges in OVA sensitised mice, AHR of comparable magnitude developed. 48 hours after the last challenge maximal Penh values at 50 mg/ml MCh were 7.38±0.72 and 6.74±0.37 for long term and short term challenges respectively, compared to 3.96±0.26 in PBS challenged controls. After short term challenges AHR resolved within one week, while it persisted for 2 weeks after long term challenges (Fig 1a). In addition, baseline Penh values in the absence of provocation were significantly increased 48 hours after OVA challenges in both treatment groups compared to mice challenged with PBS (Fig 1b). Interestingly, in contrast to AHR, baseline Penh values remained elevated for up to 6 weeks following long term challenges, while they normalised within a week after short term challenges (Fig 1b).

### Airway inflammation

**Figure 2:**
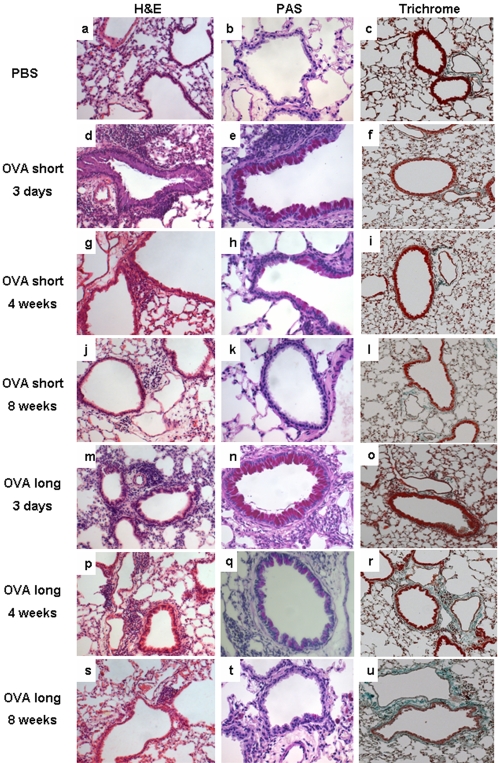
Airway inflammation, goblet cell hyperplasia and airway fibrosis following long and short term OVA challenges. Representative photomicrographs of paraffin-embedded lung sections stained with H&E (left column), Alcian-PAS (middle column) and Masson's trichrome (right column) from short term OVA challenged animals (d–l), long term OVA challenged animals (m–u) and after PBS treatment (a–c) 3 days (row 2 and 5), 4 weeks (row 3 and 6) and 8 weeks (row 4 and 7) after challenges. Magnification: 20-fold for H&E, 20-fold for Masson's trichrome, and 40-fold for Alcian-PAS stained sections.

Both short and long term airway OVA challenges induced marked airway inflammation (Fig 2d, m). Inflammation was more severe after long term than after short term challenges, with respective scores of 3.43±0.14 and 2.68±0.09, p<0.05, n = 6 per group. PBS challenged mice did not develop airway inflammation (average score 0.125±0.06, Fig 2a). All visible airways were affected by inflammation in all OVA challenged animals. Parenchymal inflammation was not detected in any of the groups. Over time, airway inflammation declined, but did not resolve completely by 8 weeks after the final challenge irrespective of the protocol used (Fig 2j, s).

**Figure 3:**
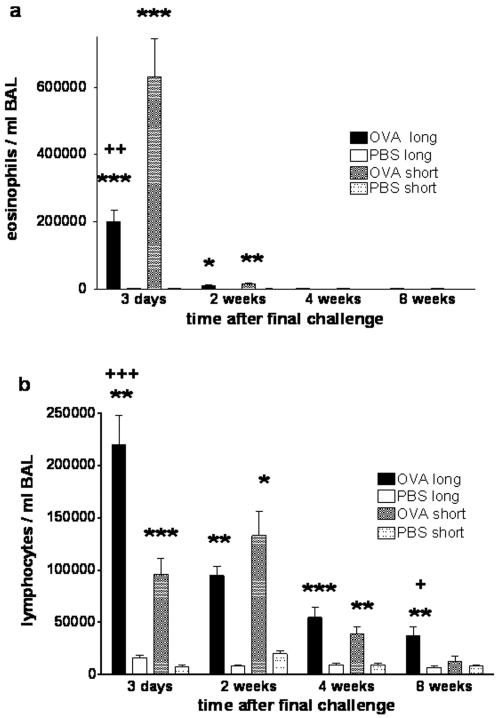
Eosinophils and lymphocytes in BAL fluid after long and short term airway challenges. Following OVA sensitisation and short or long term OVA challenges or after sham sensitisation and long or short term PBS challenges, numbers of (a) eosinophils, and (b) lymphocytes were determined in BAL fluid from 3 days to 8 weeks after final airway challenge, n≥12 per group from 5 independent experiments. Significant differences: * OVA challenges versus PBS controls, +long term versus short term OVA challenges, */+ p<0,05, **/++ p<0,01, ***/+++ p<0,001.

To characterise the quality of airway inflammation, BAL cells were analysed. 72 hours after the final challenge total cell numbers in BAL fluid were raised 6–10 fold compared to control mice without significant differences between the two challenge protocols (PBS: 124,900±18,300 cells/ml, short term OVA: *1,081,100±244,900, long term OVA: *841,800±90,200, *p<0.05 versus PBS, n≥10). BAL cell numbers remained significantly elevated (3-fold over controls) until 8 weeks after the final challenge following long term challenges, whereas they returned to baseline within 2 weeks following short term challenges (data not shown). OVA challenges resulted in eosinophilia and lymphocytosis in BAL fluid, but with striking differences between protocols (Fig 3a, b). Short term challenges resulted in very strong eosinophilia accounting for 60–70% of total BAL cells and moderate lymphocytosis representing 9% of BAL cells. In contrast, long term challenges induced marked lymphocytosis accounting for 27% of BAL cells, accompanied by moderate eosinophilia of 24% of BAL cells. Following both challenge protocols BAL eosinophilia resolved after 2 weeks, while numbers of lymphocytes decreased over time but remained significantly elevated for 4 weeks after short term and for 8 weeks after long term challenges.

### Goblet cell hyperplasia

OVA challenges in both protocols resulted in pronounced goblet cell hyperplasia 72 hours after final challenges (Fig 2e, n). Airways of PBS challenged controls did not contain PAS positive goblet cells (Fig 2b). Marked goblet cell hyperplasia was still evident 4 weeks after long term challenges (Fig 2q), but was reduced in mice challenged over 2 weeks only (Fig 2h). By 8 weeks after challenges goblet cell hyperplasia had resolved in both groups (Fig 2k, t).

### Peribronchial collagen deposition

Increased airway collagen deposition, a typical feature of airway remodelling in chronic asthma, was assessed peribronchially comparing long term to short term airway challenges. By 4 weeks after long term OVA challenges the collagen matrix around the bronchi was increased 2 to 3-fold to *8.1±4.1% (n = 9, *p<0.05) (Fig.2r) compared to short term challenges (3.1±2.4%) (Fig 2 f,i,l) and PBS controls (4.5±1.9%) (Fig 2c) and remained elevated until week 8 (*7.4±3.6) (Fig 2u). Notably, increases in collagen deposition did not develop immediately after long term challenges (3.6±1.8) (Fig 2o).

### Airway cytokine responses

**Figure 4:**
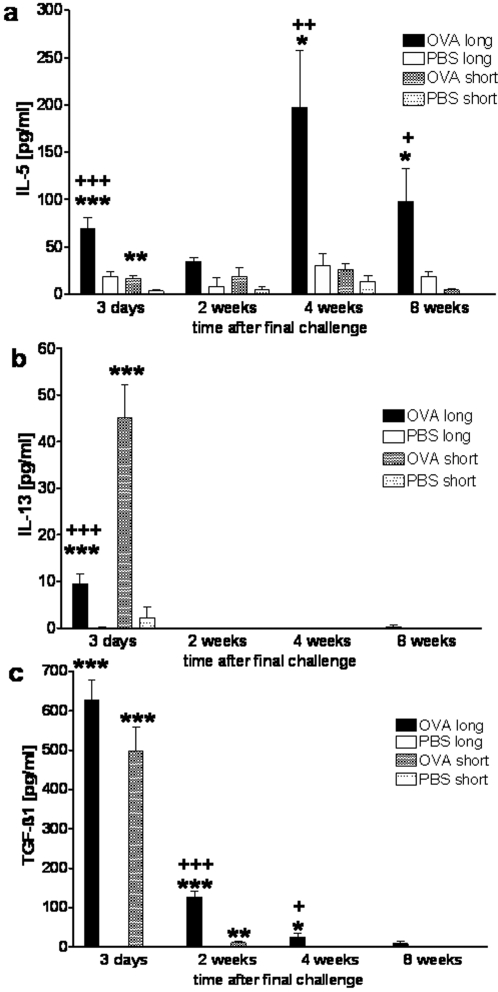
Cytokines in BAL fluid after long and short term airway challenges. Following OVA sensitisation and short or long term OVA challenges or after sham sensitisation and long or short term PBS challenges, concentrations of (a) IL-5, (b) IL-13 and (c) activated TGF-β1were measured in BAL fluid by ELISA 3 days to 8 weeks after final airway challenge, n≥15 per group from 5 independent experiments. Significant differences: * OVA challenges versus PBS controls, +long term versus short term OVA challenges, */+ p<0.05, **/++ p<0.01, ***/+++ p<0.001.

Th2 cytokines (IL-4, IL-5 and IL-13), which dominate many murine models of short term airway sensitisation, Th1cytokines (IFN-γ and IL-12), as well as IL-10, a cytokine associated with regulatory immune responses, were measured in BAL fluid by ELISA. Both short and long term OVA challenges induced increased levels of IL-5 and IL-13, however with marked differences between the two protocols (Fig 4a, b). Three days after final challenges, IL-13 dominated the response to short term challenges, while IL-5 was the dominant cytokine induced by prolonged challenges. IL-13 rapidly fell to undetectable levels by 2 weeks after challenges regardless of the protocol. IL-5 levels in contrast, persisted and even rose further 4 weeks after the final challenge in the long term challenge protocol, whereas the small increases in IL-5 after short term challenges were transient. IL-4 was only detected in minimal levels in both challenge protocols (data not shown). In addition to the Th2 cytokines, IFN-γ (up to 540 pg/ml) was induced 4 weeks after long term challenges in 25% of mice. This response failed to reach statistical significance, but IFN-γ was never detected after short term challenges. There were no significant changes in IL-10 and IL-12 levels in BAL fluid between challenge protocols (data not shown).

In addition, we assessed intracellular cytokines in lung mononuclear cells. Four weeks after long term but not short term challenges, percentages of IFN-γ producing lung cells (5.31±0.45%) were significantly elevated compared to long term PBS controls (3.64±0.55%, p<0.01, n = 10, 2 independent experiments). Similar differences 3 days and 2 weeks after long term challenges did not reach significance. Further, the percentage of IL-5 producing lung cells were significantly elevated following OVA challenges compared to PBS challenged controls and IL-5 producing lung cells were significantly more common three days and two weeks after long term OVA challenges (5.43±0.72% and 5.78±0.36%, p<0.01, n = 10) than after short term exposure (3.76±0.44% and 3.28±0.29%, both n = 10). IL-4+ lung cells were detectable after OVA challenges but there were no differences between protocols (data not shown).

### TGF-β1 in BAL fluid

In addition to the cytokines mentioned above we also assessed BAL levels of activated TGF-β1, a cytokine involved in the development of fibrosis. TGF-β1 was not detectable in control mice, but was markedly elevated 3 days after both short and long term challenges (Fig 4a). Following short term challenges, TGF-β1 levels declined quickly and became undetectable within 4 weeks. In contrast, after long term challenges TGF-β1 levels declined to a lesser degree and were still significantly increased 4 weeks after challenges and still detectable at 8 weeks.

### OVA-specific serum antibodies

Systemic OVA sensitisation with consecutive OVA challenges induced OVA-specific IgE in serum without significant differences between long and short term challenges (Table 1). OVA-specific IgG1 levels were initially significantly higher following long term challenges, but they reached comparable levels within 2 weeks after short term challenges. In addition to these antibodies which are associated with Th2 responses, prolonged airway challenges also induced robust production of OVA-specific IgG2a, an antibody found in Th1 responses. IgG2a was detectable from 3 days after challenges and throughout the 8 weeks of observation. In contrast, following short term challenges, OVA-specific IgG2a was only detected in significant amounts 8 weeks after challenges.

**Table 1:** OVA specific serum antibodies following sensitisation and short or long term challenges

	Short term OVA challenges			Long term OVA challenges		
Time post challenge	IgE [IU/ml]	IgG1 [IU/ml]	IgG2a [IU/ml]	IgE [IU/ml]	IgG1 [IU/ml]	IgG2a [IU/ml]
3 days	90±15***	34.6±11.7**	0.00±0.00	210±78*	373.9±96.7**/++	72.18±37.14*/+
2 weeks	37±10**	310.6±73.9**	0.01±0.01	60±16**	433.5±91.8**	18.39±9.20*/+
4 weeks	56±13***	138.4±64.5	4.66±4.40	96±39*	254.5±51.2***	21.6±10.11*/+
8 weeks	112±58*	92.2±57.3	40.03±11.52*	102±47*	238.4±63.9***	130.10±39.85**/+

Serum concentrations of OVA-specific antibodies post challenges were assessed by ELISA. In PBS controls no OVA-specific IgG1 or IgG2a was detected and there was a background of OVA-specific IgE of maximally 1.0 IU/ml. Significant differences: *versus respective PBS control, +versus respective time point after short term OVA challenges, */+ p<0.05, **/++ p<0.01, ***/+++ p<0,001. Means±SEM are shown, n≥9 from 3 independent experiments.

## Discussion

The pathogenic mechanisms involved in the development of chronicity in asthma are poorly understood. Recently established murine models of allergen sensitisation followed by long term allergen challenges do not only lead to impaired lung function (similar to short term challenge models) but also to airway wall remodelling, a feature typical of chronic asthma. Only a few of these studies investigated the duration of changes after the end of challenges and yielded conflicting results [5], [9], [13]. Using a murine model of systemic allergen sensitisation we compared the consequences of short and long term airway allergen challenges during eight subsequent weeks, monitoring lung function, airway inflammation, features of airway wall remodelling and immune responses.

Lung function impairment with airflow limitation is thought to be the most threatening consequence of severe asthma. It is therefore of major importance to identify mechanisms involved in the development of chronically impaired lung function. In the present model, AHR was only short lived after cessation of challenges as observed previously [9]. Recently, it has been demonstrated that AHR is sustained and even progressive while chronic allergen challenges are ongoing[12], [13]. However, persistent AHR during 8 weeks after prolonged challenges has been detected only in one model when pulmonary resistance was assessed in anaesthetised and ventilated mice challenged with MCh intravenously [5]. In addition to AHR, we observed increases in baseline Penh values early after both short and long term challenges. These resolved quickly after short term challenges, but only after 6 weeks post long term challenges. Such increases in baseline Penh, which reflect increased respiratory effort, have previously been described in mice as short lived, asthmatic late phase responses arising within hours after challenges [24]. Measuring mid-expiratory airflow in unprovoked mice, deterioration of baseline lung function has been shown during ongoing allergen challenges [13], but not after challenges as in our study.

Increases in airway collagen deposition, are thought to contribute to the development of chronic lung function impairment. This notion was supported by a report from a chronic asthma model that depletion of T-cells did not abrogate AHR [25]. The authors suggested that AHR in chronic allergen exposure is a consequence of remodelling with airway narrowing rather than of cellular inflammation. However, assessing the temporal relationship between lung function changes and collagen deposition in our study, it is striking that both AHR and reduced baseline lung function were evident before the onset of peribronchial fibrosis and that they resolved despite its persistence. This indicates that the lung function changes observed here are neither dependent on the presence of airway wall fibrosis, nor does the latter necessarily result in lung function changes. This raises the question if airway wall fibrosis is irrelevant for lung function or even if fibrosis provides a protective mechanism in asthma contributing to airway patency rather than to airflow limitation.

Goblet cell hyperplasia, another feature of airway wall remodelling, developed during challenges and thus might play a role in the induction of lung function changes. However, AHR resolved when goblet cell hyperplasia was still marked suggesting that mucus overproduction does not necessarily result in AHR. Interestingly, goblet cell hyperplasia and baseline lung function changes resolved around the same time between 4 and 8 weeks after long term challenges, suggesting a possible link.

Airway allergen challenges induced long lasting airway inflammation in our model with striking differences depending on the duration of challenges. Sustained airway lymphocytosis and moderate, short lived eosinophilia were induced by long term challenges, while short term challenges resulted in marked eosinophilia which resolved after 2 weeks. The extent of airway eosinophilia has been related to the degree of AHR in asthmatics [26], [27] and in murine models (reviewed by Hamelmann [28]). Our observations that AHR and airway eosinophilia were both of transient nature irrespective of challenge protocol support this notion. On the other hand, baseline lung function changes persisted in our model in the absence of eosinophilia, suggesting that these longer lasting changes are independent of eosinophilic inflammation.

The Th2 cytokines IL-5 and IL-13 have been implicated in the development of AHR in many models of short term allergen challenges. Following long term challenges we found persistent IL-5 production in the airways and low, transient levels of IL-13. In contrast, short term challenges were associated with strong but transient IL-13 production and low levels of IL-5. Predominant IL-13 production after short term, but not after prolonged challenges has been seen in similar models of OVA sensitisation [5], [9]. However, sensitisation to house dust mite induced expression of both IL-13 and IL-5 for several weeks after prolonged challenges [29]. In contrast to McMillan and colleagues [9], we and others [13] failed to detected IL-4 expression after long term challenges. Recent models of chronic airway allergen exposure demonstrated that the lack of IL-5 did not affect collagen deposition or AHR [18], while inhibiting BAL eosinophilia completely. IL-13 depletion prevents the development of AHR following short term challenges [30], but not in a seperate chronic asthma model, while suppressing eosinophilia and some features of remodelling [31]. These findings suggest that while Th2 cytokines may be involved in establishing airway dysfunction, they are not needed to maintain it.

In addition to Th2 responses, Th1 cytokines have been detected in severe asthma [21], [22]. In our model, a discrete transient IFN-γ response was observed in some animals 4 weeks after long term challenges, in keeping with a similar published observation [9]. The notion that Th2 and Th1 responses coexist after long term allergen challenges is strengthened further by the detection of OVA-specific IgG2a antibodies in addition to specific IgE and IgG1 antibodies in our study. IgG2a antibodies, which are associated with Th1 immune responses in the mouse, were not detected following short term challenges. Th1 responses presumably contribute to inflammation, airway remodelling and lung function changes following prolonged allergen challenges, considering that Th1 cells do not protect against allergic airway inflammation [32]. Further, IFN-γ depletion has been reported to diminish AHR and inflammatory cell accumulation in a chronic asthma model, but did not influence remodelling and eosinophil influx [31]. While the precise role of Th1 responses in chronic asthma remains to be determined, it seems that the immunology of chronic allergic airway inflammation is complex and that therapeutic strategies focussing on single cytokines only cannot be (and have not been) successful. Detrimental effects of both Th1 and Th2 responses may be antagonized by regulatory T cells, which have been shown to reduce allergen-induced AHR and inflammation in mice [33].

TGF-β1, a cytokine known to induce fibrosis, is produced by a range of airway and immune cells including a subset of regulatory T cells [34]. In our study, sustained production of TGF-β1 in the airways for up to 8 weeks after long term challenges was associated with sub-epithelial fibrosis. Marked TGF-β1 production has previously been seen during but not beyond long term OVA challenges in mice [9], [13]. In asthmatics, increases in airway TGF-β1 levels have also been demonstrated [35], [36], and TGF-β1 induces collagen synthesis in bronchial fibroblasts and transforms them to myofibroblasts [37], [38]. Considering these findings it seems likely that TGF-β1, induced by persistent airway inflammation, is an important profibrotic factor following chronic allergen exposure.

In summary, we report a mouse model of sensitisation followed by long term allergen challenges leading to sustained allergic airway inflammation, goblet cell hyperplasia, peribronchial fibrosis, and impaired lung function. The sequence in which these features arise and resolve suggests that airway wall fibrosis may not contribute to lung function impairment. The marked differences in the quality of airway inflammation and immune responses after long and short term allergen challenges may help to elucidate mechanisms responsible for chronic functional and structural changes in asthma.
